# Application of hybrid fuzzy interval-based machine learning models on financial time series — A case study of Taiwan biotech index during the epidemic period

**DOI:** 10.3389/frai.2023.1283741

**Published:** 2024-01-08

**Authors:** Hsio-Yi Lin, Bin-Wei Hsu

**Affiliations:** ^1^Department of Finance, Chien Hsin University of Science and Technology, Taoyuan, Taiwan; ^2^Department of Business Administration, Chien Hsin University of Science and Technology, Taoyuan, Taiwan

**Keywords:** hybrid fuzzy interval-based machine learning model, BPN, LSTM, Random Forest, ELM, financial time series

## Abstract

In recent years, the use of machine learning to predict stock market indices has emerged as a vital concern in the FinTech domain. However, the inherent nature of point estimation in traditional supervised machine learning models leads to an almost negligible probability of achieving perfect predictions, significantly constraining the applicability of machine learning prediction models. This study employs 4 machine learning models, namely BPN, LSTM, RF, and ELM, to establish predictive models for the Taiwan biotech index during the COVID-19 period. Additionally, it integrates the Gaussian membership function MF from fuzzy theory to develop 4 hybrid fuzzy interval-based machine learning models, evaluating their predictive accuracy through empirical analysis and comparing them with conventional point estimation models. The empirical data is sourced from the financial time series of the “M1722 Listed Biotechnology and Medical Care Index” compiled by the Taiwan Economic Journal during the outbreak of the COVID-19 pandemic, aiming to understand the effectiveness of machine learning models in the face of significant disruptive factors like the pandemic. The findings demonstrate that despite the influence of COVID-19, machine learning remains effective. LSTM performs the best among the models, both in traditional mode and after fuzzy interval enhancement, followed by the ELM and RF models. The predictive results of these three models reach a certain level of accuracy and all outperform the BPN model. Fuzzy-LSTM effectively predicts at a 68% confidence level, while Fuzzy-ELM and Fuzzy-RF yield better results at a 95% confidence level. Fuzzy-BPN exhibits the lowest predictive accuracy. Overall, the fuzzy interval-based LSTM excels in time series prediction, suggesting its potential application in forecasting time series data in financial markets to enhance the efficacy of investment analysis for investors.

## 1 Introduction

Predictability studies of stock market indices have a long history within the field of finance (Bacchetta et al., 2009; Chong et al., 2017). Researchers often analyze historical financial data as time series and establish models such as linear regression methods, Autoregressive (AR), Autoregressive Moving Average (ARMA), and Autoregressive Integrated Moving Average (ARIMA) (Li et al., 2015; Zhang et al., 2016; Kiliç and Ugur, 2018) to predict trends in stock market indices (Marszałek and Burczyński, 2014). However, due to the non-stationarity, nonlinearity, and extended lag in the time series data of stock market indices, significant patterns are often not precisely captured using conventional methods (Bildirici and Ersin, 2014; Chong et al., 2017; He et al., 2023). Consequently, in recent years, many researchers have sought to employ AI machine learning models to discover more robust predictive models for stock market indices (Sunny et al., 2020; Lim and Zohren, 2021).

Machine learning is an algorithm that uses artificial neural networks as its framework for data representation learning. The primary logic involves transforming data into a structured representation of a multi-layered neural network to facilitate learning. This enables the extraction of complex features from the data, allowing for prediction and interpretation. One advantage is that it eliminates the need for significant human effort in feature engineering and model design (Bengio et al., 2013), and it has been widely applied in recent years for extracting features from time series data. Among various machine learning models, the Back-propagation Neural Network (BPN) introduced by Rumelhart and McClelland (1986) can be considered one of the most representative and commonly used models. BPN belongs to the supervised learning framework, primarily built upon a multi-layer perceptron structure and utilizing error backpropagation. It is frequently applied in fields such as diagnostics and predictions (Rumelhart and McClelland, 1986). Qu (2003) employed BPN for infectious disease prediction, demonstrating that its predictive performance outperformed traditional multiple regression models. However, there are still studies pointing out that the effectiveness of using BPN for time series data prediction is limited. The main reason is that the learning objective of the BPN model is to establish a mapping relationship between inputs and outputs, neglecting the mutual influences among outputs. Moreover, BPN predictions are based on a sample comparison approach, rather than truly learning the relationships between time series data. Therefore, when there are mutual influences among time series, the effectiveness of computation becomes constrained (Chen et al., 2015).

Another commonly encountered machine learning model, the Recurrent Neural Network (RNN), is regarded as one of the most potent frameworks for processing temporal sequence data. The principal drawback of RNN lies in its neglect of memory capability, rendering it inadequate for capturing long-range dependencies between nodes when sequences are distantly separated. Moreover, the straightforward architecture of the RNN fails to address challenges such as the vanishing gradient problem, wherein gradients can vanish or explode due to the iterative recurrence of weights, ultimately impeding effective training. Consequently, practical instances wherein superior predictive outcomes are solely achieved via the vanilla RNN model are relatively rare. Literature frequently proposes remedies for the conventional RNN model through the design of enhanced gradient descent algorithms or the incorporation of superior activation functions within neural units. In 1997, Hochreiter and Schmidhuber (1997) introduced a groundbreaking enhancement to the RNN model known as the Long Short-Term Memory Network (LSTM). LSTM innovatively introduces memory mechanisms to augment long-term dependencies, featuring three essential steps within its neurons: forget, update, and output. This formulation substantially bolsters long-term memory performance. Additionally, LSTM partially mitigates the vanishing gradient issue encountered in RNN. Over the years, LSTM has emerged as one of the most commonly employed RNN variants. In the realm of financial time series forecasting, Di Persio and Honchar (2016) explored the suitability and effectiveness of LSTM. Selvin et al. (2017) applied LSTM along with CNN-sliding window methods for stock price prediction. Chen K. et al. (2015) highlighted the enhanced accuracy of the LSTM model in comparison to other regression models. Liu et al. (2018) specifically pointed out that LSTM-based feature extraction for time series forecasting attains an accuracy of approximately 72%, indicative of its commendable performance. Nevertheless, there remains room for refinement (Liu et al., 2018). Furthermore, LSTM encounters difficulty when handling sequences with a magnitude of 1,000 or more, and the presence of four fully connected layers (Multilayer Perceptron, MLP) within each LSTM cell can lead to computational inefficiency and time consumption as the LSTM spans longer temporal ranges or deeper network configurations. To optimize the LSTM model, scholars have proposed several enhancement strategies. Di Persio and Honchar (2016) utilized a hybrid LSTM to enhance the precision of time series predictions. Zhao et al. (2017) highlighted the superior predictive accuracy of LSTM when incorporating a time-weighted function, outperforming other deep learning models in time series forecasting.

In recent years, the Random Forest (RF) model has also been commonly employed for financial time series forecasting. It is regarded as an ensemble learning technique based on decision tree algorithms. RF employs a Bagging approach to generate multiple decision trees and then combines the predictive outcomes of these trees. The final prediction is determined through a voting mechanism, where the most frequent class is selected. However, in comparison to individual decision tree algorithms, RF exhibits stronger generalization capabilities, can handle a larger number of input variables, and is able to assess the importance of each variable (Pal, 2005). Particularly for datasets with imbalanced classes, RF can reduce errors and is less prone to overfitting issues. Lee et al. (2019) have reported an accuracy of 54.12% for stock market prediction using RF. In the analysis of stock prices within a single industry, RF demonstrates effectiveness in predicting stock prices that possess inherent randomness, thus overcoming subjective empirical judgments and the interference of emotional factors (Khaidem et al., 2016; Nana and Jiangtao, 2018). In contrast to other machine learning models, Basak et al. (2019) trained RF and XGBoost using exponential smoothing data. The accuracy of trend prediction for these two classifiers improved with an extended time window. The experimentation suggests that RF holds more advantages than XGBoost in this context. Leveraging technical indicators from the stock market, Khaidem et al. (2016) employed RF to predict stock trends. Their findings indicate that RF outperforms Support Vector Machines (SVM) and Logistic Regression (LR) in terms of obtaining more effective trend prediction results.

In addition to the aforementioned models, Huang et al. (2006) have introduced a Single-hidden Layer Feedforward Neural Network (SLFNN) known as Extreme Learning Machine (ELM). ELM has been proven to possess high learning efficiency and strong generalization capabilities, making it widely applicable to problems such as classification, regression, clustering, and feature learning (Cao et al., 2016). The number of neurons and activation function in ELM must be regulated, as the input weights and hidden layer biases are fixed during its application. These characteristics contribute to ELM's reputation for achieving enhanced generalization performance with rapid learning. Cheng et al. (2009) have elegantly demonstrated ELM's superiority over SVM in predicting petroleum reservoir permeability. Huang et al. (2011) have successfully implemented ELM for regression and classification tasks across various domains. Over the past decade, ELM has consistently shown its advantages over traditional techniques in the realm of stock market forecasting (Sun et al., 2014; Li et al., 2016). Due to the fact that traditional feedforward neural networks (such as BPN) require manual configuration of a significant number of network training parameters, ELM stands out for its simplicity and ease of use. Unlike BPN, ELM only requires the setting of the network's structure and doesn't necessitate the adjustment of other parameters. The weights from the input layer to the hidden layer are determined in a single random iteration and do not need further tuning during execution. Similarly, the weights from the hidden layer to the output layer are determined by solving a linear system of equations, generally contributing to improved computational speed.

However, from a statistical perspective, if the predicted values of the aforementioned supervised machine learning models are only point estimates with binary outcomes (such as binary classification or single-point prediction), it will lead to the problem of the estimated probability of perfect correctness approaching zero. This is due to the fact that in continuous random variables, single points hold no probability value. Therefore, the point estimation prediction method greatly restricts the usability of machine learning models (Lowe and Zapart, 1999). In contrast to point forecasting, probabilistic forecasting describes the variation of the value by providing outputs in terms of probabilistic density function, confidential intervals of the distribution. It can better describe the uncertainty of values (Gan et al., 2017). In reality, the best predictions should include estimated probability distribution intervals for a future time period to better align with real-world situations. In related studies, Quantile regression is utilized in Liu et al. (2017) to generate multiple forecasting results. In Yang et al. (2006), Liu et al. (2017), and Xie et al. (2017) simulation of historical-error distribution was implemented to convert point loads into intervals. Zadeh (1965) introduced Fuzzy Logic in his publication “Information and Control,” aiming to utilize fuzzy phenomena to address the reasoning model of uncertainty in the real world. Fuzzy logic has since been widely applied in artificial intelligence fields such as automatic control, pattern recognition, and decision analysis. Whether fuzzy logic can be applied to predictive models in machine learning is a topic worth exploring. Ballings et al. (2015), in comparing traditional models and integrated models in the machine learning domain, demonstrated that integrated models perform better than single models in predicting financial data based on time series. This study will build upon the existing BPN, LSTM, RF, and ELM models, which are commonly used machine learning models. Initially, point estimation predictions for stock price indices will be computed. Subsequently, by integrating the Gaussian membership function (MF) of fuzzy theory, interval calculations will be performed to develop a fuzzy interval-based machine learning model. Empirical analysis will further investigate whether these models can achieve more accurate predictions of stock price indices. It is anticipated that the outcomes of this study will enhance the practicality and predictive capabilities of machine learning models in real-world scenarios.

In addition, due to the outbreak of the COVID-19 epidemic in recent years, various countries have implemented city closures and restricted crowd activities, which has had a significant impact on the economy and financial markets. Frequent phenomena such as stock market crashes, plummeting commodity prices, and declining global demand have created greater uncertainty for investors. Therefore, during the epidemic period, whether in the economic field or social field, many linear or machine learning prediction model-related research Generated in large quantities, for example: Wu et al. (2022) once used a time series prediction model to predict half-hourly electricity demand in Victoria. Zhao et al. (2023) once constructed a deep learning framework, combining time autocorrelation with Spatially correlated combination, reflecting the impact of neighboring cities and historical data on air quality during COVID-19. Cui et al. (2023) propose a deep learning framework with a COVID-19 adjustment for electricity demand forecasting. In summary, when the market is faced with the noise and interference of the epidemic, what impact will the machine learning model have on the forecast accuracy of the Taiwan stock market index? It is also one of the topics that this study is interested in exploring.

This study employs the highly representative dataset compiled by the Taiwan Economic Journal (TEJ), specifically the “M1722 Listed Biotechnology and Medical Care Index” (hereinafter referred to as the Taiwan TEJ Biotech Index), as empirical data to represent the performance of Taiwan's listed biotechnology and medical care stock market industry. Furthermore, due to the severe stock market fluctuations caused by the COVID-19 pandemic (Baret et al., 2020; Uddin et al., 2021), which exhibit dynamics different from non-pandemic periods, the empirical period of this study is set to the outbreak of the COVID-19 pandemic (from January 2020 to the end of June 2022). The aim is to understand the extent to which the accuracy of machine learning model predictions is affected when the stock market experiences significant turmoil due to pandemic-related disturbances. In summary, this study has three main objectives: (1) To establish predictive models for the Taiwan biotech index after the COVID-19 outbreak using four machine learning models: BPN, LSTM, RF, and ELM. (2) To integrate fuzzy theory to modify the existing point estimation approach of machine learning models and thus develop a fuzzy interval-based machine learning model, while comparing it with traditional point estimation models. (3) To understand whether machine learning models are suitable for predicting stock indices when the stock market faces significant disturbances and substantial fluctuations (such as during the outbreak of the COVID-19 pandemic).

## 2 Materials and methods

### 2.1 Variables

This study focuses on the “Taiwan TEJ Biotechnology Index” as the research subject, with the closing prices of the Taiwan biotechnology index during the COVID-19 outbreak period as the research object. The primary data source is the TEJ database. The study aims to compare 8 machine learning models: BPN, LSTM, RF, ELM, as well as fuzzy interval-based BPN (fuzzy-BPN), fuzzy interval-based LSTM (fuzzy-LSTM), fuzzy interval-based RF (fuzzy-RF), and fuzzy interval-based ELM (fuzzy-ELM), in terms of their predictive accuracy.

Initially, literatures were gathered to collect various variable data used for index prediction. These variables include Taiwan index data, international index data, futures prices, sentiment indicators, macroeconomic analysis, and 23 other variables. The study then employed factor analysis to identify significant variables affecting the Taiwan biotechnology index during the COVID-19 outbreak period. Subsequently, a model was established using MATLAB to predict the closing prices of the Taiwan TEJ Biotechnology Index, thereby validating the feasibility of the proposed research methodology.

The research framework consists of 9 steps, which are elaborated as follows:

1. Data Collection: Involves the collection of various variable indicators, including seven technical indicators, five variables related to Taiwan's biotechnology and healthcare sector's net buying and selling, as well as trading volume, 8 variables related to Taiwan and international index market trends, one sentiment indicator, one futures price index, and one macroeconomic analysis, total 23 variable indicators (see Table 1).
2. Data Preprocessing: Due to variations in trading holidays for international stock market, if trading data cannot be obtained due to market closures or other reasons on certain days, the entire set of data will be removed in advance.
3. Removal of Ineffective Variables: Confirmatory Factor Analysis (CFA) is employed to select appropriate variables as input indicators for the research model. Ineffective variables are eliminated to enhance the predictive accuracy of the model.
4. Normalization: The data is subjected to normalization, scaled to a range between 0 and 1.
5. Model Construction: Eight machine learning models are individually established using the TEJ listed biotechnology and healthcare sector index (see Table 2).
6. Setting Training and Testing Parameters: Divide the data in a 7:3 ratio, setting it as training data and testing data, respectively.
7. Train the model.
8. Validate Predictive Results: If the predicted results are not as expected, repeat steps 5 to 7.
9. Model Comparison: Compare the predictive accuracy of MODEL1 to MODEL8 from step (5), and perform a comparison of predictive capabilities using indicators such as the Mean Absolute Percentage Error (MAPE).

**Table 1:** Variables in this study.

**Category**	**Variables**
Technical indicators	MA_5, MA_20, K_9, D_9, RSI_6, RSI_12, MACD_9
Biotech and medical sector indicators	Listed biotech and medical sector turnover rate, biotech and medical sector mutual fund net buy/sell, biotech and medical sector proprietary trading net buy/sell, biotech and medical sector foreign investor net buy/sell, biotech and medical sector equity-to-debt ratio.
Composite index	Taiwan Weighted Index, U.S. Dow Jones Industrial Average, U.S. S and P 500 Index, U.S. Nasdaq Biotechnology Index, Shanghai Composite Index, Hong Kong Hang Seng Index, South Korea Composite Index, Japan Nikkei 225 Index.
News-based index	VIX Fear Index
Futures index	CRB Index
Macroeconomic analysis	Business Cycle Indicators

**Table 2:** Machine learning models in this study.

**Model**	**Model**
BPN	MODEL 1
LSTM	MODEL 2
RF	MODEL 3
ELM	MODEL 4
Fuzzy-BPN	MODEL 5
Fuzzy-LSTM	MODEL 6
Fuzzy-RF	MODEL 7
Fuzzy-ELM	MODEL 8

### 2.2 Data processing

The research data were sampled from the month of the first confirmed COVID-19 case in Taiwan in January 2020 until the end of June 2022, excluding market holidays for Taiwan and international index markets. A total of 513 samples were collected, with 359 samples used for learning and 154 samples used for testing. This study conducted confirmatory factor analysis on 23 variables. Factors with eigenvalues >1 were extracted (Kaiser, 1960), followed by rotation using the maximum variance rotation method. The results indicated that nine variables should be excluded from the initial 23 variables. These variables are: economic policy signals, South Korea composite index, Shanghai composite index, biotech and medical stock-to-asset ratio, CRB index, VIX panic index, US Nasdaq biotechnology NBI index, listed biotech and medical stock turnover rate, and mutual fund net buying and selling. The remaining 14 variables (MA_5, MA_20, K_9, D_9, RSI_6, RSI_12, MACD_9, biotech and medical proprietary net buying and selling, biotech and medical foreign net buying and selling, Taiwan weighted index, US Dow Jones Industrial Average, US S&P 500 index, Hong Kong Hang Seng index, Nikkei 225 index) were selected as the final input variable indicators for this study, explaining a total of 80.48% of the variance. After adjusting the aforementioned indicator variables, their suitability was tested with a Kaiser-Meyer-Olkin measure of 0.679, exceeding the recommended threshold of 0.6 (Tabachnick and Fidell, 1996), and a Bartlett's sphericity test approximate chi-square distribution value of 9668.408 with a *p*-value of 0.000 for 91 degrees of freedom. These values suggest that the data of these 14 input variable indicators are suitable for subsequent analysis. To standardize the residuals between data points, the study normalized the variable data. Normalization was performed using the sampled data and the maximum value (X_max_) and minimum value (X_min_) within a range. Depending on whether the initial value of the variable was ≥0 or had negative values, equations (1) and (2) were used separately to obtain the normalized value (X_nom_). This normalized value serves as the input variable data for the deep learning model in this study.

If the initial values of the variables are all ≥0:


(1) $$\begin{array}{l} X_{nom}=\frac{X-X_{\min}}{X_{\max}-X_{\min}} \end{array}$$

If the initial values of the variables have negative values:


(2) $$\begin{array}{l} X_{nom}=\frac{X}{\max|X|} \end{array}$$

(Denominator is the maximum absolute value of X).

### 2.3 Machine learning model

#### 2.3.1 BPN

**Figure 1:**
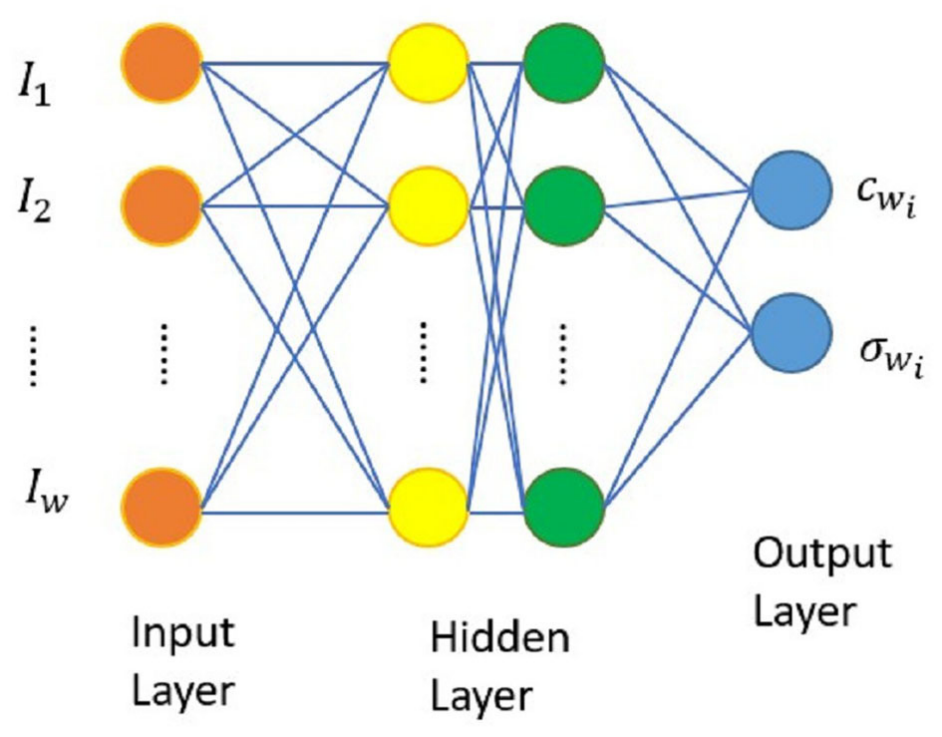
Architecture diagram of BPN (Chen and Lin, 2007).

BPN, introduced by Rumelhart and McClelland (1986), is a supervised learning feedforward multilayer network architecture, incorporating the concept of hidden layers and bias weights. The network architecture is illustrated in Figure 1. It consists of 3 main layers: the input layer, hidden layer, and output layer, each containing multiple processing units. Units in different layers are interconnected through threshold values and weight values. Input variables are transmitted from the input layer to the hidden layer, computed, and then propagated to the output layer.

After comparing the differences between actual values and output variables, the results are propagated back to the hidden layer. Based on this, the weight values of the connecting links are adjusted. This iterative training process employs the steepest descent method. Whenever a training sample is input, the network adjusts the weights by an amount Δ*w*_*ij*_ [expressed using equations (3) and (4)], continuing until the error converges under predetermined conditions.


(3) $$\begin{array}{l} \Delta w_{ij}=-\eta \frac{\partial E}{{\partial w}_{ij}} \end{array}$$

η represents the learning rate, which serves the purpose of controlling the magnitude of each step in the steepest descent method to minimize the error function. E represents the error function.


(4) $$\begin{array}{l} E=\frac{1}{2}\sum {\left(T_{j}-A_{j}\right)}^{2} \end{array}$$

*T*_*j*_ represents the target output value of the j-th unit in the output layer.

*A*_*j*_ represents the inferred output value of the j-th unit in the output layer.

#### 2.3.2 LSTM

**Figure 2:**
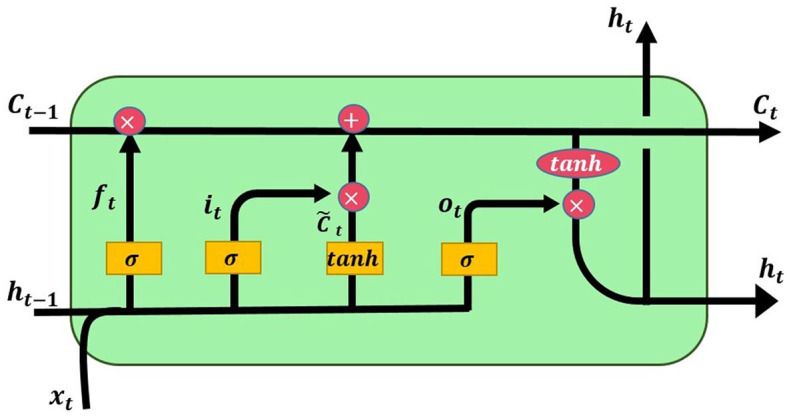
Architecture diagram of LSTM.

LSTM is a model derived from recurrent neural networks (RNN) that incorporates memory units. It was introduced by Hochreiter and Schmidhuber (1997). LSTM primarily employs the Sigmoid activation function and dot product operations to control the switches of three gates (input gate, output gate, forget gate), determining which data can be stored in the memory unit. The input gate mainly controls whether input values flow into the memory unit; the output gate regulates whether data computed through the Tanh activation function should be output; the forget gate's main purpose is to decide whether the stored information from the previous time step should be forgotten or retained in the memory unit. Due to its memory units, LSTM is capable of recording longer information compared to RNN and addresses the issue of poor performance in long-term memory of RNN. Hence, it is more frequently used than RNN. Figure 2 shows the architecture of the LSTM model (Liu and Wei, 2022).

The function of LSTM at time t is given by equations (5) to (11).


(5) $$\begin{array}{l} f_{t}=\sigma_{g}\left(x_{t}w_{f}+h_{t-1}U_{f}+b_{f}\right) \end{array}$$

(6) $$\begin{array}{l} i_{t}=\sigma_{g}\left(x_{t}w_{i}+h_{t-1}U_{f}+b_{i}\right) \end{array}$$

(7) $$\begin{array}{l} o_{t}=\sigma_{g}\left(x_{t}w_{o}+h_{t-1}U_{o}+b_{o}\right) \end{array}$$

(8) $$\begin{array}{l} i_{t}=\sigma_{g}\left(x_{t}w_{i}+h_{t-1}U_{f}+b_{i}\right) \end{array}$$

(9) $$\begin{array}{l} \tilde{c_{t}}=\sigma_{h}\left(x_{t}w_{c}+h_{t-1}U_{c}+b_{c}\right) \end{array}$$

(10) $$\begin{array}{l} c_{t}=f_{t}\times c_{t-1}+i_{t}\times \tilde{c_{t}} \end{array}$$

(11) $$\begin{array}{l} h_{t}=o_{t}\times \sigma_{h}\left(c_{t}\right) \end{array}$$

*x*_*t*_ represents the input data at time t

*h*_*t*_ represents the output of the hidden layer at time t

*w*_*f*_, *w*_*o*_, *w*_*c*_, *w*_*i*_, *U*_*f*_, *U*_*o*_, *U*_*c*_, *U*_*i*_ represents the weight function

*b*_*f*_, *b*_*o*_, *b*_*c*_, *b*_*t*_ are bias parameters

σ_*g*_ is sigmoid function, σ_*h*_ is tanh function

*f*_*t*_, *i*_*t*_, *o*_*t*_ are the forget, input, and output gates, respectively.

#### 2.3.3 ELM

ELM is a feedforward neural network devised by Professor Guang-Bin Huang from Nanyang Technological University in Singapore. Unlike conventional artificial neural networks such as BPN that necessitate the configuration of numerous network training parameters, ELM solely requires the specification of the network's structure, omitting the necessity for additional parameters. Hence, it has gained renown for its straightforwardness and user-friendliness (Cao et al., 2016). In this investigation, we embrace the structure of a single-layer feedforward neural network (SLFN) for ELM. This structure comprises an input layer, a hidden layer, and an output layer. The output function FL of the hidden layer is delineated as equation (12):


(12) $$\begin{array}{l} f_{L}=\sum_{i=1}^{l}\beta_{i}h_{i}\left(x\right)=h\left(x\right)\beta \end{array}$$

Within the equation, the symbol *x* signifies the input variable, while l denotes the number of nodes in the hidden layer. β corresponds to the output weight, and *h*(*x*) embodies the activation function responsible for transforming data from the input layer into the feature space of ELM. This expression is depicted as equation (13):


(13) $$\begin{array}{l} h\left(x\right)=G\left(a_{i},b_{i},x\right) \end{array}$$

In the provided equation, the variables *a*_*i*_ and *b*_*i*_ represent feature mapping parameters, often referred to as node parameters. Specifically, *a*_*i*_ denotes the input weight or input weights in this context. This investigation utilizes the widely used Sigmoid function, as depicted in equation (14):


(14) $$\begin{array}{l} G\left(a_{i},b_{i},x\right)=\frac{1}{1+\exp\left(a\cdot x+b\right)} \end{array}$$

The goal of training a neural network with a single hidden layer revolves around the minimization of output errors. By undergoing the process of learning and training, we can derive the values of β that lead to the achievement of minimal and distinct error.

#### 2.3.4 RF

Breiman (2001) introduced RF in 2001. RF operates based on the concept of ensemble learning, where it amalgamates several decision trees to create a more resilient learning model. This, in turn, addresses the challenge of overfitting, leading to enhanced predictive accuracy within the domain of machine learning.

Breiman's definition of RF, delineated in equation (15), depicts an assembly of tree-like structures that collectively shape a classifier:


(15) $$\begin{array}{l} \left\{h\left(x,k\right),\;k=1,\ldots \right\} \end{array}$$

In this definition, {k} represents an array of independently and identically distributed random vectors. This conglomerate of classifiers converges through their amalgamation, as illustrated in equation (16):


(16) $$\begin{array}{l} h_{1}\left(x\right),\;h_{2}\left(x\right),\;\ldots ,h_{k}\left(x\right) \end{array}$$

By creating the training set in a random manner from the probability distributions of random vectors X and Y, the margin function is established as outlined in equation (17):


(17) $$\begin{array}{l} mg\left(X,Y\right)\\ =\alpha v_{k}I\left(h_{k}\left(X\right)=Y\right)-max_{j\neq Y}{av}_{k}\alpha v_{k}I\left(h_{k}\left(X\right)=j\right) \end{array}$$

Here, *I* represents the indicator function utilized for the accurate classification of X and Y. The magnitude of the margin function directly corresponds to an elevated correct classification score. The generalization error is precisely defined as depicted in equation (18):


(18) $$\begin{array}{l} PE^{*}=P_{X,Y}\left(mg\left(X,Y\right)<0\right) \end{array}$$

In this context, X and Y stand as representations of probabilities. The effectiveness of the RF model is commonly evaluated based on the subsequent considerations:

1. A more robust growth trajectory for each tree corresponds to an enhanced overall performance of the forest.
2. Improved independence and reduced correlation among individual trees within the forest lead to superior classification performance.
3. The quantity of decision trees stands as the sole parameter for RF execution and serves as the pivotal determinant for achieving the RF model with the minimum error.

### 2.4 Hybrid fuzzy interval-based machine learning model

Supervised machine learning models and deep learning models share a common characteristic: the predicted values of the output variable are point estimates. Despite the advantages of highly predictive interpretability and low-error precision in machine learning models, the drawback of single-point probabilistic estimation still exists. To address this, this study attempts to propose a fuzzy membership function to enhance and intervalize machine learning models, aiming to mitigate the shortcomings of point estimation while retaining the ability of machine learning models to handle dynamic and complex data.

The traditional approach to modeling financial time-series data heavily relied on normal distributions until 1963 when Mandelbrot (1963) challenged this norm. He noticed leptokurtosis in the empirical distributions of price changes and suggested using symmetric stable distributions to account for this excess kurtosis. Subsequent developments by researchers such as Ali and Giaccotto (1982), Kon (1984), Bookstaber and McDonald (1987), and Barinath and Chaterjee (1988) advanced the use of various non-normal distributions for modeling financial data. Despite these advancements in characterizing financial data with non-normal distributions, there remains a lack of techniques to fully explain their distribution.

Addressing this gap, this paper introduces a fuzzy-interval architecture to enhance machine learning models, referred to as Fuzzy machine learning models. These models utilize fuzzy sets, defined by a membership function (MF), to overcome the limitations of single-point predictions inherent in traditional machine learning models. Specifically, the Gaussian MF, a common assumption in normal distribution, is adopted, characterized by two parameters: the center {c, σ} equation (19):


(19) $$\begin{array}{l} f\left(x;c,\sigma \right)\;=\;\frac{1}{\sqrt{2\;\pi \;\sigma }}e^{-\frac{1}{2}{\left(\frac{x\;-\;c}{\sigma }\right)}^{2}} \end{array}$$

where is the Gaussian MF's center and σ determines MF's width. In this paper, indicates the mean of n-days returns or indices, σ denotes the standard deviation of n-day returns or indices, and the MF of fuzzy-interval is also decided completely by and σ. The Gaussian MF in this approach is essentially an extension of the normal distribution, a fundamental concept in probability theory. Central to our methodology is the placement of the fuzzy-interval MF around a central point *c*, allowing for a range of variance that includes 1.68σ (representing a 95% probability) and 1.96σ (accounting for a 99% probability). This strategic inclusion of 1.68σ and 1.96σ within the interval significantly enhances traditional machine learning models by addressing their inherent limitation of relying solely on single-point predictions. By adopting this approach, the model not only adheres to the principles of Gaussian distribution but also substantially improves prediction accuracy by accommodating a wider range of outcomes, thereby rectifying the shortcoming of single-point forecasting prevalent in conventional machine learning models.

**Figure 3:**
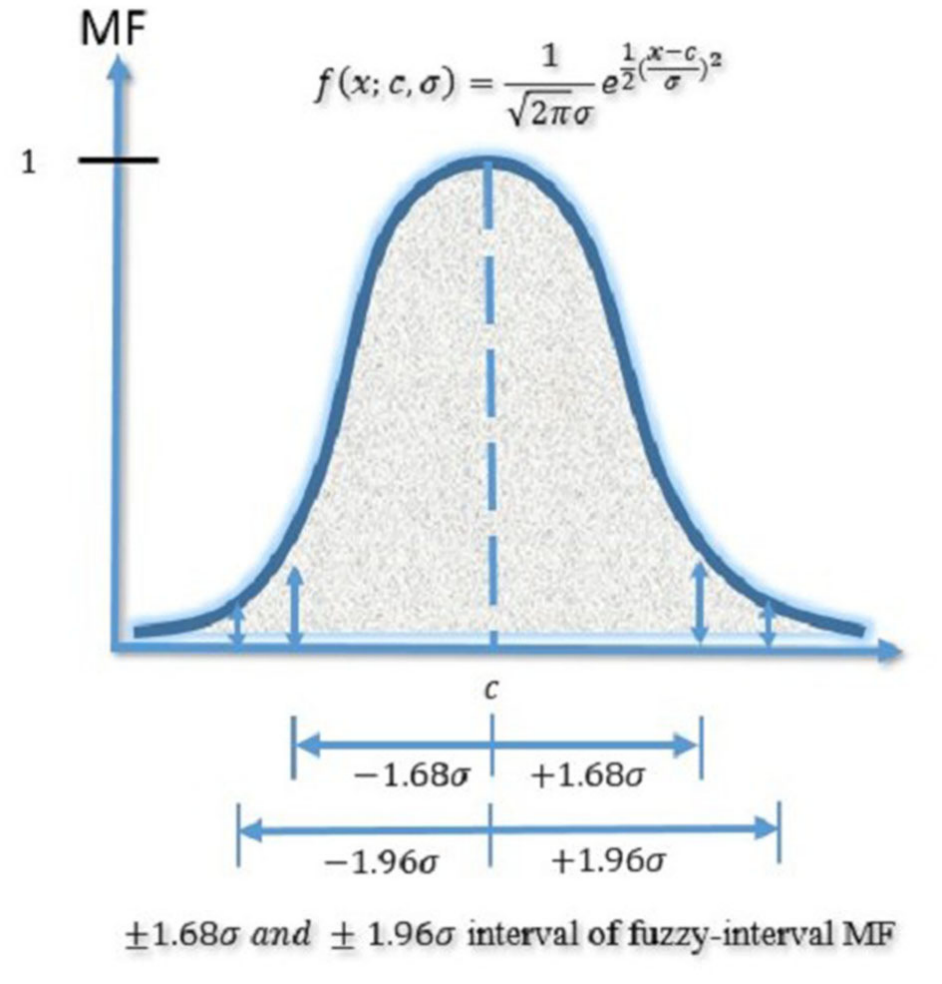
Gaussian MF of the fuzzy-interval approach.

**Figure 4:**
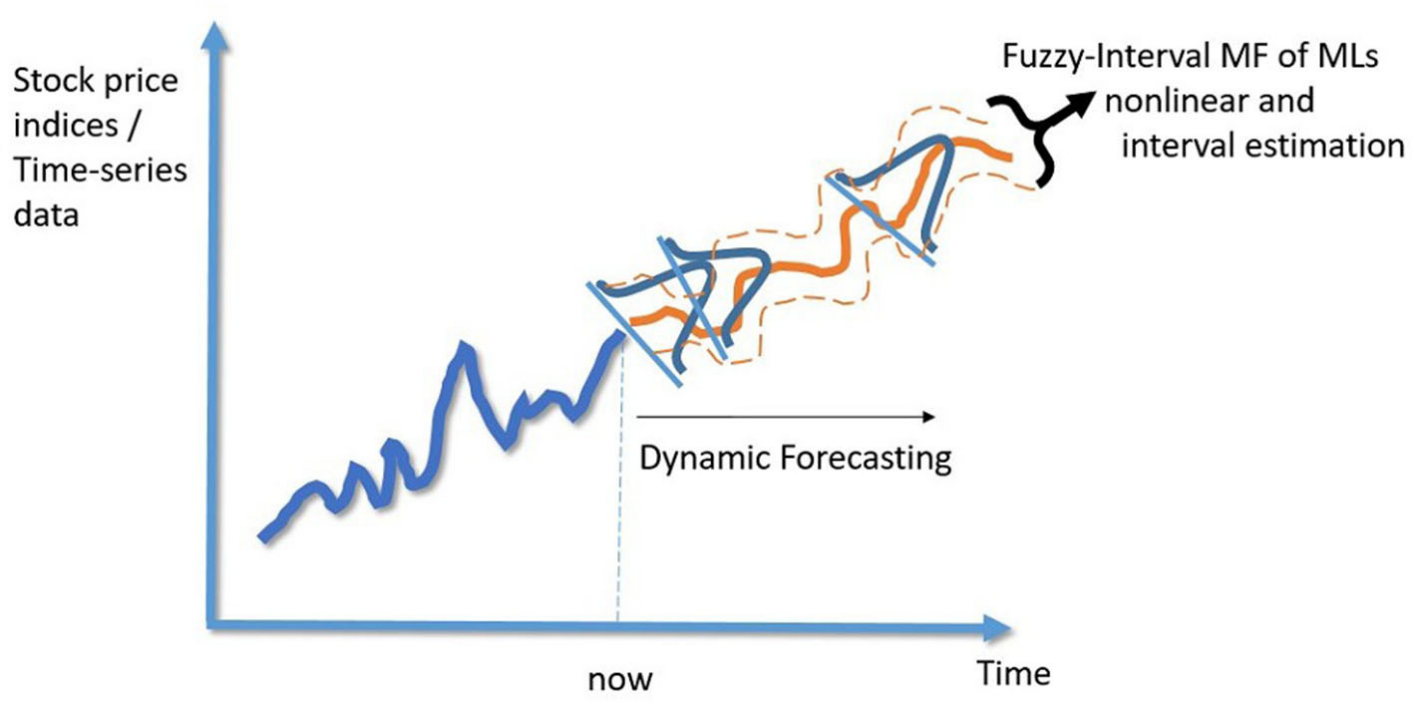
Conceptual diagram of Gaussian membership functions (MFs).

Figure 3 illustrates the Gaussian MF of the fuzzy-interval approach. Building on this foundation, the paper endeavors to identify the parameters and σ using machine learning models. Figure 4 will demonstrate the framework for generating the fuzzy-interval MF, allowing for the retention of the nonlinear characteristics of machine learning models while simultaneously enhancing them by addressing their single-point prediction constraints. This novel framework is termed Fuzzy machine learning models in our study.

In the context of this study, *c* represents the center of the Gaussian MFs, while σ governs the width of these MFs. Within the scope of this paper, *c* denotes the average value of the weekly Taiwan Biotechnology Index, while σ signifies the standard deviation of the same index on a weekly basis. Moreover, the characteristics of the fuzzy-interval MF are entirely determined by the values of *c* and σ. It's worth noting that the Gaussian Membership Function is a straightforward extension of the normal distribution employed in probability theory. In the case of the fuzzy-interval MF, its center is aligned with *c*, and its spread around *c* is defined by adding and subtracting 1.68 or 1.96 times the value of σ, representing the corresponding confidence intervals.

The fuzzy-machine learning models proposed in this study utilize the characteristics of financial time series data, taking the dynamic N-day average and standard deviation to determine the center and width of the interval. In application, it does not require extensive mathematical derivations and computations to complete the dynamic estimation interval.

### 2.5 Evaluation index

The evaluation indices used employed in this research for gauging the effectiveness of the trained models consist of RMSE (Root Mean Square Error), MAPE (Mean Absolute Percentage Error), and MAE (Mean Absolute Error). The formulas are presented as follows equations (20–22):


(20) $$\begin{array}{l} RMSE=\sqrt{\frac{1}{n}\times \sum_{i=1}^{n}{\left(\hat{Y_{i}}-Y_{i}\right)}^{2}} \end{array}$$

(21) $$\begin{array}{l} MAPE=\frac{100\%}{n}\sum_{i=1}^{n}|\frac{\hat{Y_{i}}-Y_{i}}{Y_{i}}| \end{array}$$

(22) $$\begin{array}{l} MAE=\frac{\sum |{\hat{Y_{i}}-Y}_{i}|}{n} \end{array}$$

*Y*_*i*_ : Actual value

Ŷ_*i*_: Predicted output value from the network

n: Number of test examples

Among the aforementioned indicators, RMSE is a statistical metric that measures the difference between predicted and actual values, and commonly used to assess model accuracy. It calculates the mean of the squared prediction errors and then takes the square root, providing a measure in the same units as the original data. A smaller RMSE indicates higher accuracy of the predictive model. However, as it is influenced by the data range, it's suitable for comparing predictive errors of specific variables among different models. MAPE is a relative measure that determines the degree of difference between estimated and actual values, independent of unit influences. It calculates the absolute percentage error for each predicted value and then takes the average of these errors. A lower MAPE indicates higher accuracy in the predictive model. Generally, a MAPE% value below 10 is considered highly accurate, between 10 and 20 signifies good accuracy, between 20 and 50 suggests reasonable accuracy, and values exceeding 50 are deemed inaccurate (Lewis, 1982). MAE, a metric used to assess the error of a predictive model, representing the sum of absolute differences between target and predicted values, measures the average length of prediction errors without considering their direction. It ranges from 0 to positive infinity. A lower MAE indicates smaller errors in the predictive model, meaning less average difference between predicted and actual values.

Furthermore, when evaluating interval-based ML, this study also employs the Accuracy (ACC) metric to assess model prediction performance. ACC gauges the accurate prediction ratio of the model and is calculated as shown in equation (23).


(23) $$\begin{array}{l} ACC=\frac{t}{n} \end{array}$$

t: Number of the actual values that fall within the predicted CIs.

n: Total number of test examples.

## 3 Results

In this study, a total of 359 training examples and 154 testing examples were used for the eight models, resulting in a total of 513 examples. The parameter settings and empirical performance of the BPN, LSTM, RF, and ELM models are detailed as follows.

### 3.1 Empirical analysis of BPN

In this study, the BPN model was implemented using Matlab 2021 software. Regarding the determination of BPN model parameters, Zhang et al. (1998) indicated that the most commonly used number of neural network layers is 1 or 2, with usually 1 hidden layer achieving highly effective prediction performance. Yoon et al. (1993) found through empirical research that a 2-layer hidden layer configuration provides better predictive capabilities for time series. Therefore, this study will test parameters with 1 to 2 hidden layers.

Furthermore, for determining the number of nodes in the hidden layers, Davies (1994) stated that the suitable number of nodes for each hidden layer can only be found through a trial-and-error approach. Lawrence and Petterson (1991), on the other hand, recommended that the number of nodes in each hidden layer should be tested based on 50 to 75% of the sum of input and output variables. Therefore, this study intends to test a range of approximately 7 to 12 nodes, considering the total of 15 nodes (14 input variables and 1 output variable) as 50 to 75% of the range, using a trial-and-error method. As for the learning rate, Freeman and Skapura (1992) explained that the learning rate of an artificial neural network should be <1 to achieve optimal learning state and convergence. Therefore, this study plans to test a learning rate of 0.5 and set the training cycles to 1,000 or terminate the learning process if the RMSE has converged for the BPN model. Regarding the empirical execution of training and testing the BPN model in this study, the evaluation was conducted using RMSE, MAE, and MAPE metrics, and the results are summarized in the Table 3.

**Table 3:** Empirical Performance of BPN.

**Hidden layer parameters**	**Data set**	**RMSE**	**MAE**	**MAPE**
10	Training	1.6292	1.1643	1.3060%
	Testing	1.1983	0.8613	1.2709%
12	Training	1.4818	1.0725	1.3371%
	Testing	1.1131	0.8303	1.2252%
7^*^10	Training	1.7902	1.4344	1.4509%
	Testing	1.4707	1.2094	1.7845%
7^*^12	Training	1.7450	1.2701	1.3992%
	Testing	1.3223	1.0784	1.5912%
10^*^10	Training	1.6006	1.1983	1.3767%
	Testing	1.2500	0.9607	1.4176%
10^*^12	Training	1.6787	1.2231	1.3844%
	Testing	1.3392	1.0107	1.4914%

Red and bold text indicate the best performance of BPN model.

After experimentation through trial and error, it was found that the optimal model configuration is with a single hidden layer and 12 nodes.

### 3.2 Empirical analysis of LSTM

The empirical implementation of the LSTM model in this study was also conducted using Matlab2021 software. Currently, there is no definitive standard for setting the parameters of the LSTM model, and adjustments are often determined through a trial-and-error approach (Chen et al., 2019). In this study, the number of hidden layers was tested between 2 and 3, and the number of nodes in each hidden layer was adjusted from 60 to 256. The range for the number of iterations was set between 100 and 1,000, and the dropout rate for hidden layer weights ranged from 0.2 to 0.5. The learning rate was tested within the range of 0.005 to 0.01, and the learning rate decay factor was set to 0.02. These parameter ranges were consolidated from various literature sources. The parameter values tested through the trial-and-error method are summarized in the following Table 4.

**Table 4:** Trial-and-error Parameter List for the LSTM Model.

**Model**	**Hidden layer**	**Weight loss**	**Epoch time**	**Learning rate**
LSTM 1	60^*^180^*^60	0.2/0.3/0.2	300	0.005
LSTM 2	128^*^256^*^64	0.3/0.3/0.3	300	0.005
LSTM 3	256^*^256^*^64	0.5/0.5/0.5	1,000	0.01
LSTM 4	128^*^128	0.2/0.2	300	0.005
LSTM 5	128^*^256	0.3/0.3	1,000	0.005
LSTM 6	180^*^180	0.5/0.5	100	0.01

Through implementation in Matlab2021, the results for the LSTM model are presented in the Table 5.

**Table 5:** Performance of LSTM model validation.

**Model**	**Data set**	**RMSE**	**MAE**	**MAPE**
LSTM 1	Training	0.6889	0.5315	0.8023%
	Testing	1.5719	1.2889	1.9601%
LSTM 2	Training	1.9659	1.6023	2.4702%
	Testing	2.3904	1.8512	2.9331%
LSTM 3	Training	4.5501	3.2104	4.9538%
	Testing	4.8382	3.4790	5.5118%
LSTM 4	Training	0.6200	0.4804	0.7268%
	Testing	0.7646	0.5811	0.8989%
LSTM 5	Training	0.4009	0.3088	0.4637%
	Testing	0.6486	0.4880	0.7603%
LSTM 6	Training	1.2653	0.9750	1.5053%
	Testing	1.5645	1.1268	1.7844%

Red and bold text indicate the best performance among all the models.

From the Table 5, it can be observed that based on the performance evaluation using RMSE, MAPE, and MAE values, the optimal parameter configuration for the LSTM model is the 5th set (LSTM 5) model.

### 3.3 Empirical analysis of ELM and RF

The ELM model, as a single hidden layer neural network, offers advantages such as not requiring the setup of numerous parameters and having strong learning capabilities, compared to the traditional BPN. In this study, the parameter settings for the ELM model are as follows: a single hidden layer with 30 nodes, determined through trial and error to achieve good convergence. The activation function used is the commonly used Sigmoid function, and the remaining parameters are set to their default values. For the RF classification model in this study, the number of decision trees is set to 20 using the TreeBagger function. RF is specified to operate in regression mode. The feature selection method is set to “curvature,” which selects split points based on the curvature of features. The other parameters are set to their default values in the program.

Based on the aforementioned execution results, the RMSE, MAE, and MAPE values of the empirical models in this study have all converged to reasonable standards. Among the 4 machine learning tools, the LSTM model exhibits the best convergence state. The differences in error values between ELM and RF are not significant, and the RMSE, MAE, and MAPE values of these 3 models are superior to those of the BPN model.

### 3.4 Empirical summary: fuzzy-BPN, fuzzy-LSTM, fuzzy-ELM, and fuzzy-RF

In summary, to address the limitation of traditional “single-point without probability” point estimation, this study proposes the incorporation of Fuzzy Gaussian Membership Function for interval estimation improvement, aiming to enhance the accuracy of predicting actual values. Under the assumption that confidence intervals are extended by 1 standard deviation for 68%, 1.96 standard deviations for 95%, and 2.58 standard deviations for 99%, the predictive results are presented in the **Table 7**.

**Figure 5:**
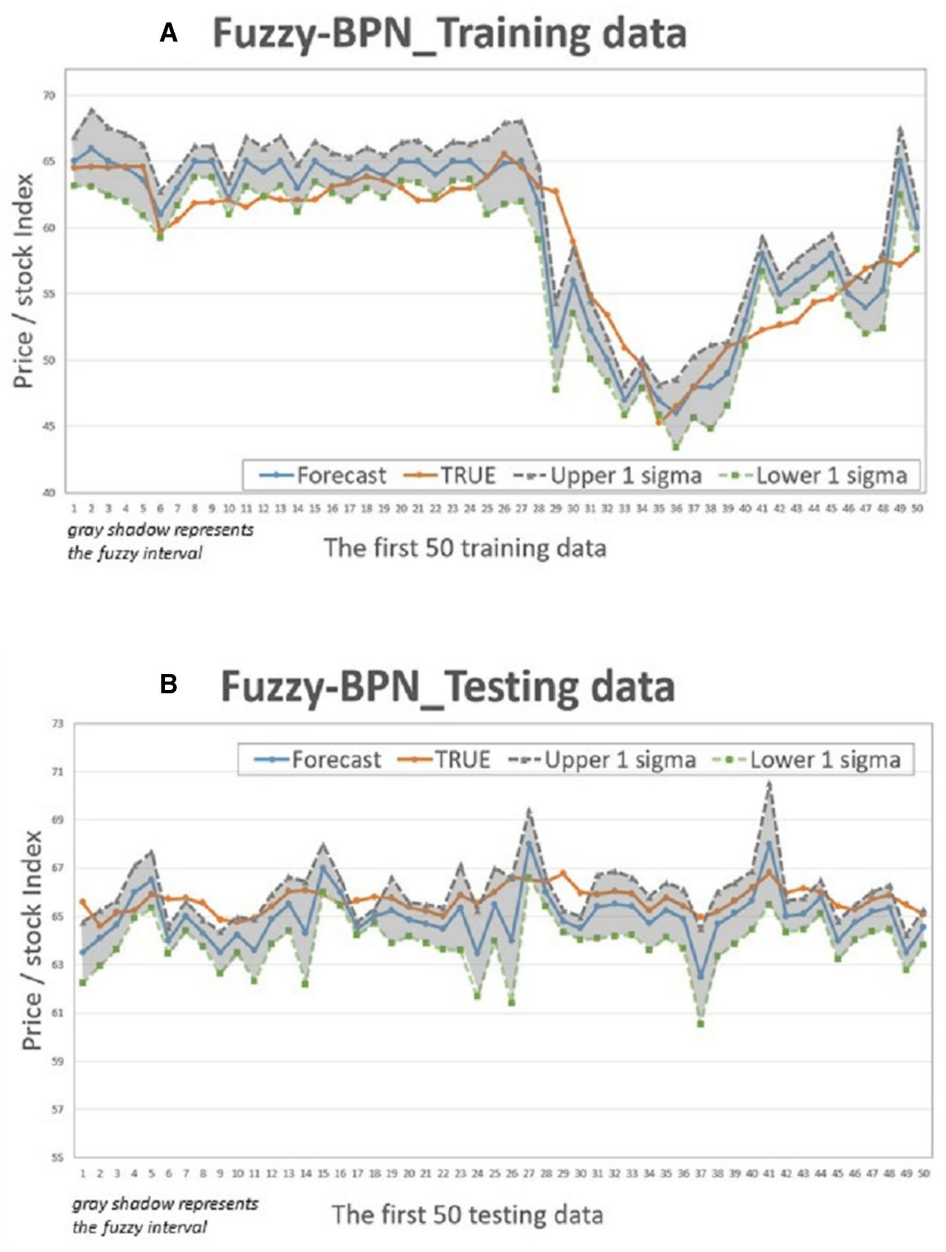
Fuzzy-BPN Interval chart (for the first 50 data). **(A)** Training data; **(B)** Testing data.

**Figure 6:**
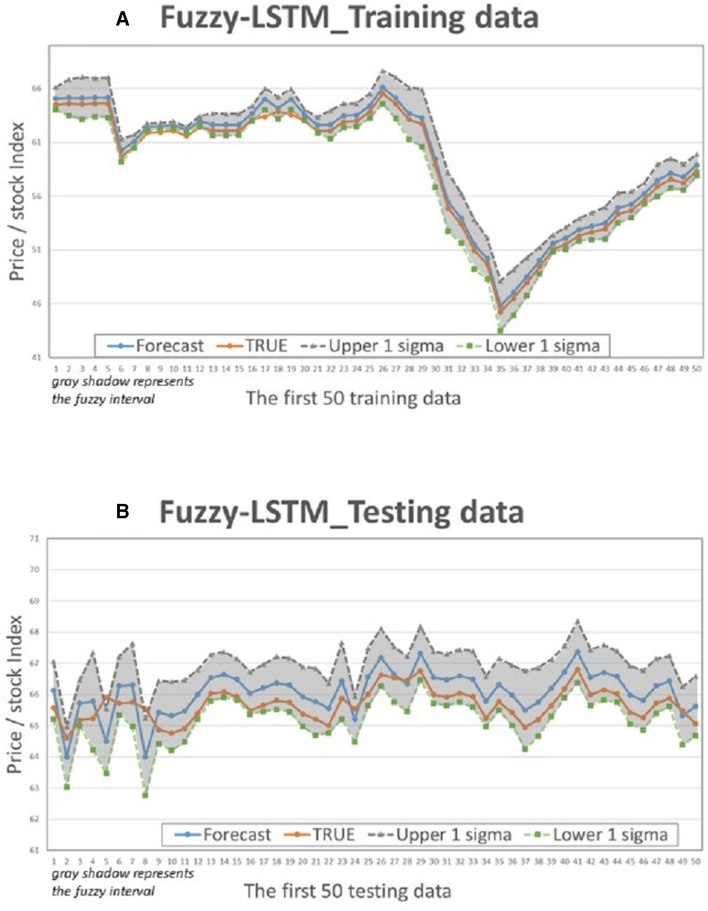
Fuzzy-LSTM Interval chart (for the first 50 data). **(A)** Training data; **(B)** Testing data.

**Figure 7:**
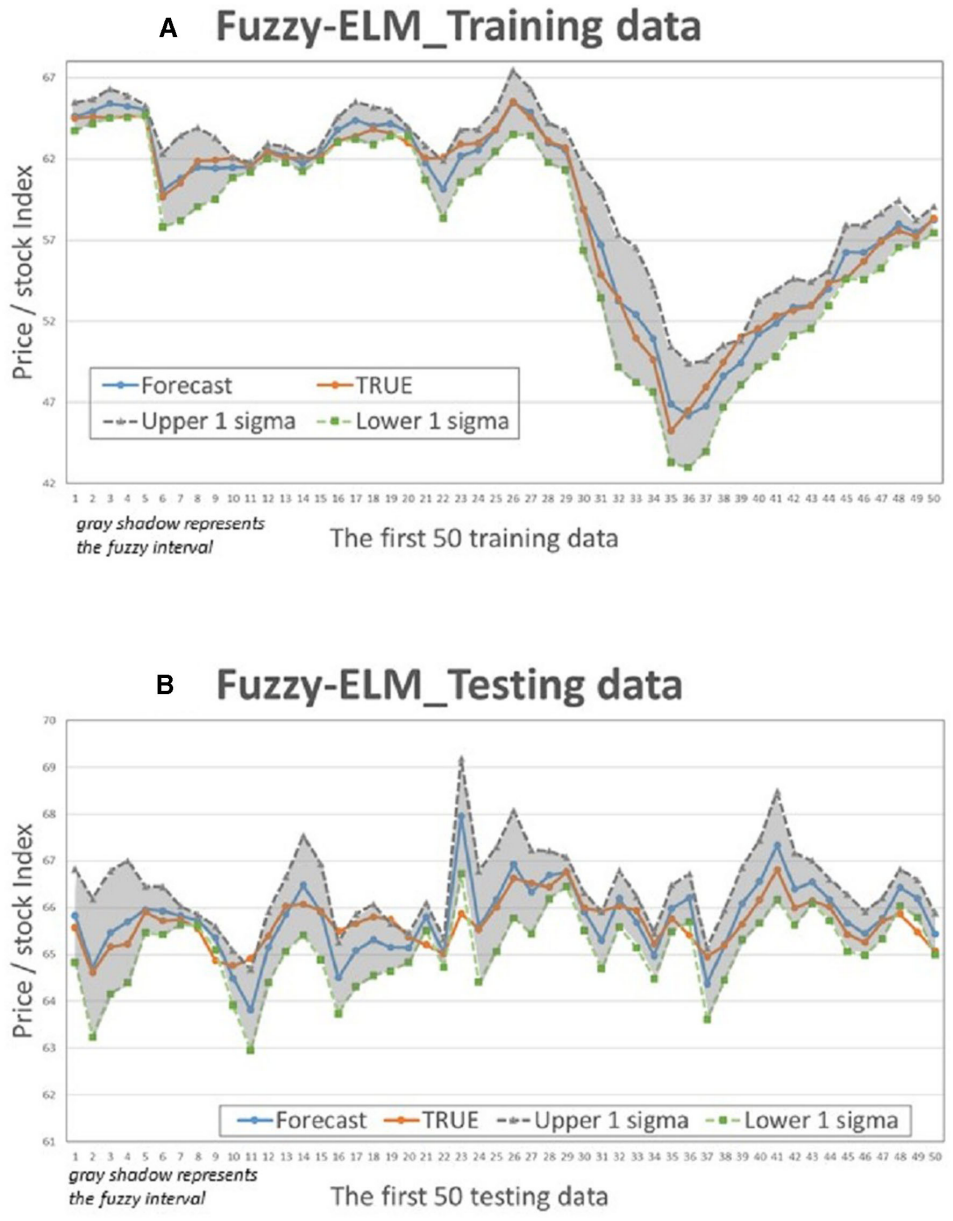
Fuzzy-ELM Interval chart (for the first 50 data). **(A)** Training data; **(B)** Testing data.

**Figure 8:**
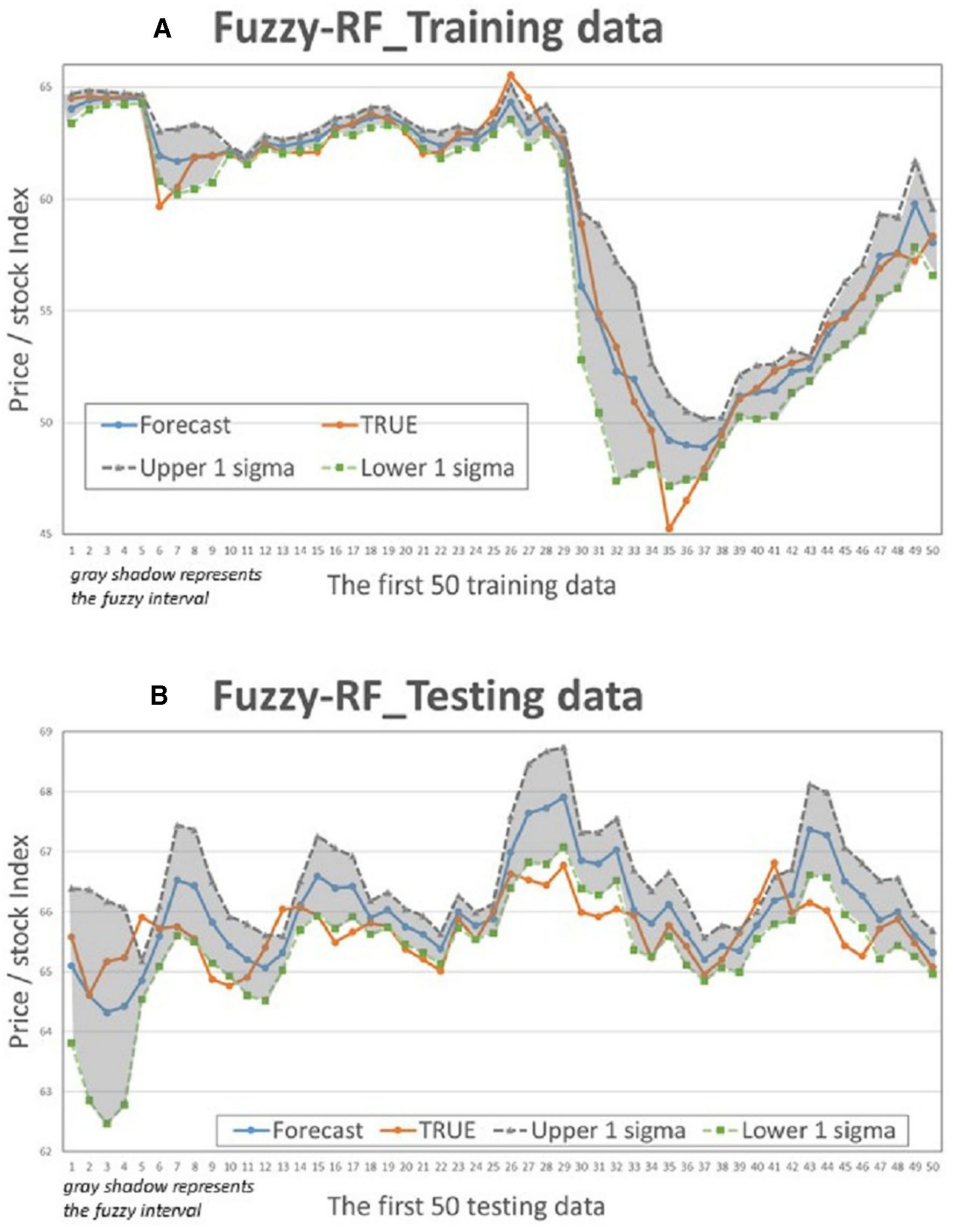
Fuzzy-RF Interval chart (for the first 50 data). **(A)** Training data; **(B)** Testing data.

This study compares the actual values with the intervals formed based on the predictions and standard deviation multiples of the 4 models to determine if the actual values fall within the predicted intervals. Figures 5–8 illustrate the predictive values, upper and lower bounds, as well as the actual qualitative chart (for the first 50 data points) of the four fuzzy interval-based machine learning models during both the training set and testing set phases. Thus, Tables 6, 7, it is evident that the LSTM model exhibits the best convergence state in both training and testing examples among all machine learning models in this study. It achieves the smallest errors in terms of RMSE, MAE, and MAPE, followed by the RF, ELM, and BPN models. Additionally, as shown in Table 7, compared to other models, the Fuzzy-LSTM, in terms of ±1σ, ±1.68σ, and ±1.96σ, interval levels, maintains an effective predictive accuracy of over 97%. In contrast, the Fuzzy-BPN shows the least ideal prediction results, with a maximum accuracy of only 70.56%. The empirical results demonstrate the superior performance of the Fuzzy-LSTM model. At the 99% confidence level of the prediction interval, all models except Fuzzy-BPN achieve an accuracy of at least 85%, including Fuzzy-LSTM, Fuzzy-ELM, and Fuzzy-RF. Notably, the Fuzzy-LSTM model even achieves a 100% accuracy rate in predicting the actual values of the test dataset. It is clear that as the interval size and coverage increase, the predictive accuracy also improves. The second-best performing model is Fuzzy-ELM, which achieves an accuracy rate of nearly 76% at the 68% confidence interval level. In summary, the Hybrid Fuzzy interval-based machine learning models (LSTM, ELM, RF) indeed are capable of effectively capturing the time-series characteristics of stock price data in financial market time series and accurately predicting their values.

**Table 6:** Performance of ELM, RF, BPN, and LSTM models.

**Evaluation index**	**Data set**	**ELM**	**RF**	BPN^*^	LSTM^*^
RMSE	Training	0.6007	0.6110	1.488	0.4009
	Testing	1.0014	1.0605	1.1131	0.6486
MAE	Training	0.4623	0.4180	1.0725	0.3088
	Testing	0.8146	0.8782	0.8303	0.4880
MAPE	Training	0.7017%	0.6432%	1.3371%	0.4637%
	Testing	1.2517%	1.3433%	1.2252%	0.7603%

^*^Means that the performance of the model is based on the optimal parameter combination identified through trial and error.

**Table 7:** Performance of hybrid fuzzy interval-based machine learning model.

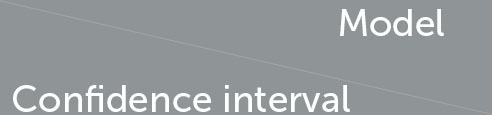	**Fuzzy-BPN**		**Fuzzy-LSTM**		**Fuzzy-ELM**		**Fuzzy-RF**	
**Training**	**Testing**	**Training**	**Testing**	**Training**	**Testing**	**Training**	**Testing**
68% (± 1σ)	41.67%	39.22%	**97.18%** ^*^	**97.00%** ^*^	82.78%^*^	76.47%	71.94%	50.33%
95% (± 1.68σ)	56.11%	46.41%	**99.44%** ^*^	**99.33%** ^*^	95.83%^*^	94.12%^*^	91.67%^*^	80.39%^*^
99% (± 1.96σ)	70.56%	63.40%	**99.27%** ^*^	**100%** ^*^	97.78%^*^	97.39%^*^	95.00%^*^	85.62%^*^

Red and bold text indicate the best performance among the 4 models. ^*^The performance of prediction accuracy is higher than 80%.

## 4 Discussion

This study presents an interval estimation principle to address the limitations of traditional machine learning models' point estimates and aims to enhance the capability of time series prediction. The summarized findings are as follows:

1. Both the traditional BPN and LSTM models require trial-and-error methods to find optimal parameter combinations. After comparing with literature recommendations and trial-and-error adjustments, the error values of the LSTM model in this study are consistently lower than those of the BPN model. On the other hand, the ELM and RF models require less trial and error to adjust parameters, resulting in faster training and testing processes. Although their error values are larger than those of the LSTM model, their predictive results are acceptable and outperform the BPN model. Overall, the results suggest that the LSTM model is more suitable for predicting time series data of the biotech and medical index during the COVID-19 period.
2. Despite the impact of the COVID-19 pandemic, the volatility of Taiwan's biotech index did not experience significant fluctuations compared to industries like the service or tourism sector. It remained relatively stable, allowing for effective learning by machine learning models despite the relatively short sampling period.
3. As described in (2), empirical evidence suggests that the Fuzzy-LSTM model with a 68% confidence level estimation can provide effective and reasonable predictions. The Fuzzy-ELM and Fuzzy-RF models perform better with a 95% confidence level estimation, while the Fuzzy-BPN model exhibits the lowest predictive accuracy among all models.
4. The proposed hybrid fuzzy interval LSTM model (LSTM, ELM, RF) in this study achieves high predictive accuracy for time series data. It implies that they are indeed capable of effectively capturing the time-series characteristics of stock price data in financial market time series and accurately predicting their values. Future applications of this approach in predicting time series data in other financial markets are recommended, as it could enhance the effectiveness of investment analysis measures for relevant financial decision-makers.
5. When making investment forecasts with financial data, investors not only focus on potential profits but also pay close attention to the risk management of their investment portfolios. If an inadvertent investment error leads to losses, the ability to reasonably estimate the maximum possible loss can make investors more willing to fund investment activities. An improved interval estimation machine learning tool can incorporate Value at Risk (VaR) (Jorion, 1996) to estimate the maximum potential loss for effective risk management. This represents a suggested direction for future research in this thesis.

## Data availability statement

The original contributions presented in the study are included in the article/supplementary material, further inquiries can be directed to the corresponding author.

## Author contributions

H-YL: Conceptualization, Data curation, Formal analysis, Methodology, Software, Writing – original draft, Writing – review & editing. B-WH: Data curation, Investigation, Project administration, Validation, Visualization, Writing – original draft, Writing – review & editing.

## References

[B1] Ali M. N. Giaccotto C. (1982). The identical distribution hypothesis for stock market prices: location and scale shift alternatives. J. Am. Stat. Assoc. 77, 19–28. 10.1080/01621459.1982.10477762

[B2] Bacchetta P. Mertens E. van Wincoop E. (2009). Predictability in financial markets: what do survey expectations tell us? J. Int. Money Finance 28, 406–426. 10.1016/j.jimonfin.2008.09.001

[B3] Ballings M. Van den Poel D. Hespeels N. Gryp R. (2015). Evaluating multiple classifiers for stock price direction prediction. Expert Syst. Appl. 42, 7046–7056. 10.1016/j.eswa.2015.05.013

[B4] Baret S. Celner A. O'Reilly M. Shilling M. (2020). COVID-19 Potential Implications for the Banking and Capital Markets Sector. London: Deloitte Center for Financial Services.

[B5] Barinath S. G. Chaterjee S. (1988). On measuring skewness and elongation in common stock return distributions, the case of market index. J. Business 61, 451–472. 10.1086/296443

[B6] Basak S. Kar S. Saha S. Khaidem L. Roy Dey S. (2019). Predicting the direction of stock market prices using tree-based classifiers. North Am. J. Econ. Finance 47, 552–567. 10.1016/j.najef.2018.06.013

[B7] Bengio Y. Courville A. C. Vincent P. (2013). Representation learning: a review and new perspectives. IEEE Trans. Pattern Anal. Mach. Intell. 35, 1798–1828. 10.1109/TPAMI.2013.50 23787338

[B8] Bildirici M. Ersin Ö. Ö. (2014). Nonlinearity volatility and fractional integration in daily oil prices: smooth transition autoregressive ST-FI (AP) GARCH models. Rom. J. Econ. Forecast 3, 108–135.

[B9] Bookstaber R. M. McDonald J. B. (1987). A general distribution for describing security price returns. J. Business 60, 401–424. 10.1086/296404

[B10] Breiman L. (2001). Random Forests. Mach. Learn. 45, 5–32. 10.1023/A:1010933404324

[B11] Cao J. W. Zhang K. Luo M. X. Yin C. Lai X. P. (2016). Extreme learning machine and adaptive sparse representation for image classification. Neural Netw. 81, 91–102. 10.1016/j.neunet.2016.06.001 27389571

[B12] Chen A. P. Lin H. Y. (2007). “Exchange rates forecasting using a hybrid fuzzy and neural network model,” in Proceedings of IEEE Symposium on Computational Intelligence and Data Mining (CIDM) (Honolulu), 758–763. 10.1109/CIDM.2007.368952

[B13] Chen C.-C. Kuo C. Kuo S.-Y. Chou Y.-H. (2015). “Dynamic normalization BPN for stock price forecasting,” *in Proceedings of the 2015 IEEE International Conference on Systems, Man, and Cybernetics* (Hong Kong), 2855–2860. 10.1109/SMC.2015.497

[B14] Chen K. Zhou Y. Dai F. (2015). “ALSTM-based method for stock returns prediction: a case study of China stock market,” in Proceedings of the 2015 IEEE International Conference on Big Data (Big Data) (Santa Clara, CA), 2823−2824. 10.1109/BigData.2015.7364089

[B15] Chen Z. H. Chen Y. L. Chang W. Y. Tsai C. W. (2019). A hybrid classification algorithm for intrusion detection system. Commun. CCISA 25, 14–27.

[B16] Cheng G. J. Cai L. Pan H. X. (2009). “Comparison of extreme learning machine with support vector regression for reservoir permeability prediction,” in Proceedings of the 2009 International Conference on Computational Intelligence and Security (CIS) (Beijing: IEEE), 173–176. 10.1109/CIS.2009.124

[B17] Chong E. Han C. Park F. C. (2017). Deep learning networks for stock market analysis and prediction: methodology, data representations, and case studies. Expert Syst. Appl. 83, 187–205. 10.1016/j.eswa.2017.04.030

[B18] Cui Z. Wu J. Lian W. Wang Y. -G. (2023). A novel deep learning framework with a COVID-19 adjustment for electricity demand forecasting. Energy Rep. 9, 1887–1895. 10.1016/j.egyr.2023.01.019

[B19] Davies P. C. (1994). Design issues in neural network development. Neurovest J. 5, 21–25.

[B20] Di Persio L. Honchar O. (2016). Artificial neural networks architectures for stock price prediction: comparisons and applications. Int. J. Circ. Syst. Signal Process. 10, 403–413.

[B21] Freeman J. A. Skapura D. M. (1992). Neural Networks Algorithms, Applications, and Programming Techniques. CA, USA: Addison-Wesley.

[B22] Gan D. Wang Y. Zhang N. Zhu W. (2017). Enhancing short-term probabilistic residential load forecasting with quantile long-short-term memory. J. Eng. 14, 2622–2627. 10.1049/joe.2017.0833

[B23] He K. Yang Q. Ji L. Pan J. Zou Y. (2023). Financial time series forecasting with the deep learning ensemble model. Mathematics 11:1054. 10.3390/math11041054

[B24] Hochreiter S. Schmidhuber J. (1997). Long short-term memory. Neural. Comput. 9, 1735–1780. 10.1162/neco.1997.9.8.1735 9377276

[B25] Huang G. B. Zhou H. Ding X. Zhang R. (2011). Extreme learning machine for regression and multiclass classification. IEEE Trans. Syst. Man. Cybern. B Cybern. 42, 513–529. 10.1109/TSMCB.2011.2168604 21984515

[B26] Huang G. B. Zhu Q. Y. Siew C. K. (2006). Extreme learning machine: theory and applications. Neurocomputing 70, 489–501. 10.1016/j.neucom.2005.12.126

[B27] Jorion P. (1996). Risk - measuring the risk in value at risk. Financ. Anal. J. 52, 47–56. 10.2469/faj.v52.n6.2039

[B28] Kaiser H. F. (1960). The application of electronic computers to factor analysis. Educ. Psychol. Meas. 20, 141–151. 10.1177/001316446002000116

[B29] Khaidem L. Saha S. Dey S. R. (2016). Predicting the Direction of Stock Market Prices using Random Forest.

[B30] Kiliç D. K. Ugur Ö. (2018). Multiresolution analysis of SandP500 time series. Ann. Oper Res. 260, 197–216. 10.1007/s10479-016-2215-3

[B31] Kon S. J. (1984). Models of stock returns - a comparison. J. Finance 39, 147–165. 10.1111/j.1540-6261.1984.tb03865.x

[B32] Lawrence M. Petterson A. (1991). Getting Started with Brain Maker: Neural Network Simulation Software User's Guide and Reference Manual/Introduction to Neural Networks and Disk. MA, USA: California Scientific Software.

[B33] Lee T. K. Cho J. H. Kwon D. S. Sohn S. Y. (2019). Global stock market investment strategies based on financial network indicators using machine learning Techniques 117, 228–242. 10.1016/j.eswa.2018.09.005

[B34] Lewis E. B. (1982). Control of body segment differentiation in drosophila by the bithorax gene complex. Embryo. Dev. 1, 383–417. 10.1007/978-1-4419-8981-9_15 7111279

[B35] Li P. Jing C. Liang T. Liu M. Chen Z. Guo L. (2015). “Autoregressive moving average modeling in the financial sector,” in Proceedings of the 2nd International Conference on Information Technology Computer and Electrical Engineering (ICITACEE) (Semarang), 68–71. 10.1109/ICITACEE.2015.7437772

[B36] Li X. Xie H. Wang R. Cai Y. Cao J. Wang F. . (2016). Empirical analysis: stock market prediction via extreme learning machine. Neural Comput. Appl. 27, 67–78. 10.1007/s00521-014-1550-z

[B37] Lim B. Zohren S. (2021). Time series forecasting with deep learning: a survey. Philos. Trans. R. Soc. 379, 202–209. 10.1098/rsta.2020.0209 33583273

[B38] Liu B. Nowotarski J. Hong T. Weron R. (2017). Probabilistic load forecasting via quantile regression averaging on sister forecasts. IEEE Trans. Smart Grid. 8, 730–737. 10.1109/TSG.2015.2437877

[B39] Liu D. Wei A. (2022). The performance of hybrid artificial neural network models for option pricing during financial crises. J. Data Sci. 14, 1–18. 10.6339/JDS.201601_14(1)0.0001

[B40] Liu S. Liao G. Ding Y. (2018). “Stock transaction prediction modeling and analysis based on LSTM,” in Proceedings of the IEEE Conference on Industrial Electronics and Applications (ICIEA) (Wuhan), 2787–2790. 10.1109/ICIEA.2018.8398183

[B41] Lowe D. Zapart K. (1999). Point-wise confidence interval estimation by neural networks: a comparative study based on automotive engine calibration. Neural Comput. Appl. 8, 77–85. 10.1007/s005210050009

[B42] Mandelbrot B. B. (1963). The variation of certain speculative prices. J. Business 36, 394–419. 10.1086/294632

[B43] Marszałek A. Burczyński T. (2014). Modeling and forecasting financial time series with ordered fuzzy candlesticks. Inf. Sci. 273, 144–155. 10.1016/j.ins.2014.03.026

[B44] Nana L. Jiangtao Q. (2018). Research on A-share stock rise and fall prediction based on Random Forest. J. Shanghai Univ. Technol. 267–273.

[B45] Pal M. (2005). Random Forest classifier for remote sensing classification. Int. J. Remote Sens. 26, 217–222. 10.1080/01431160412331269698

[B46] Qu B. (2003). The research of the effect and forecast of meteorological factors on epidemic situation of common infectious diseases in drought area (Master's thesis). TaiChung: China Medical University.

[B47] Rumelhart D. E. McClelland J. L. (1986). Parallel Distributed Processing, Explorations in the Microstructure of Cognition. Vol. 1: Foundations. Cambridge, MA: MIT Press. 10.7551/mitpress/5236.001.0001

[B48] Selvin S. Vinayakumar R. Gopalakrishnan E. Menon V. K. Soman K. (2017). “Stock price prediction using LSTM RNN and CNN-sliding window model,” in Proceedings of the 2017 International Conference on Advances in Computing, Communications and Informatics (ICACCI) (Udupi), 1643–1647. 10.1109/ICACCI.2017.8126078

[B49] Sun F. Toh K. A. Romay M. G. Mao K. (2014). Extreme Learning Machines: Algorithms and Applications. Berlin: Springer International Publishing. 10.1007/978-3-319-04741-6

[B50] Sunny M. A. I. Maswood M. M. S. Alharbi A. G. (2020). “Deep learning-based stock price prediction using LSTM and bi-directional LSTM model,” in Proceedings of the 2nd Novel Intelligent and Leading Emerging Sciences Conference (NILES) (Giza), 87–92.

[B51] Tabachnick B. G. Fidell L. S. (1996). Using Multivariate Statistics (3rd edn.). Giza: Harper Collins.

[B52] Uddin M. Chowdhury A. Anderson K. Chaudhuri K. (2021). The effect of COVID−19 pandemic on global stock market volatility: can economic strength help to manage the uncertainty? J. Bus. Res. 128, 31–44. 10.1016/j.jbusres.2021.01.061 36540352 PMC9754760

[B53] Wu J. Levi N. Araujob R. Wang Y. -G. (2022). An evaluation of the impact of COVID-19 lockdowns on electricity demand. Electr. Power Syst. Res. 216:109015. 10.1016/j.epsr.2022.109015

[B54] Xie J. Hong T. Laing T. Kang C. (2017). On normality assumption in residual simulation for probabilistic load forecasting. IEEE Trans. Smart Grid. 8, 1046–1053. 10.1109/TSG.2015.2447007

[B55] Yang W. Kang C. Xia Q. Liu R. Tang T. Wang P. (2006). Short-term probabilistic load forecasting based on statistics of probability distribution of forecasting errors. Autom. Electr. Power Syst. 19, 11. 10.1109/TPWRS.2005.860937

[B56] Yoon Y. Swales G. Margavio T. M. (1993). A comparison of discriminant analysis versus artificial neural networks. J. Oper. Res. Soc. 44, 51–60. 10.1057/jors.1993.6

[B57] Zadeh L. A. (1965). Fuzzy sets. Inf. Control 8, 338–353. 10.1016/S0019-9958(65)90241-X

[B58] Zhang G. Patuwo B. E. Hu M. Y. (1998). Forecasting with artificial neural networks: the state of the art. Int. J. Forecast. 14, 35–62. 10.1016/S0169-2070(97)00044-7

[B59] Zhang G. Zhang X. Feng H. (2016). Forecasting financial time series using a methodology based on autoregressive integrated moving average and Taylor expansion. Expert. Syst. 33, 501–516. 10.1111/exsy.12164

[B60] Zhao Z. Rao R. Tu S. (2017). “Time-weighted LSTM model with redefined labeling for stock trend prediction,” in Proceedings of the IEEE 29th International Conference on Tools with Artificial Intelligence (ICTAI) (Boston, MA), 1210–1217. 10.1109/ICTAI.2017.00184

[B61] Zhao Z. Wu J. Cai F. Zhang S. Wang Y. -G. (2023). A hybrid deep learning framework for air quality prediction with spatial autocorrelation during the COVID-19 pandemic. Sci. Rep. 13, 1015. 10.1038/s41598-023-28287-8 36653488 PMC9848720

